# The Neurochemical Characterization of Parasympathetic Nerve Fibers in the Porcine Uterine Wall Under Physiological Conditions and After Exposure to Bisphenol A (BPA)

**DOI:** 10.1007/s12640-019-00013-1

**Published:** 2019-02-20

**Authors:** Rytel Liliana, Gonkowski Slawomir, Janowski Tomasz, Wojtkiewicz Joanna, Pomianowski Andrzej

**Affiliations:** 1 Department of Internal Disease with Clinic, Faculty of Veterinary Medicine, University of Warmia and Mazury, ul. Oczapowskiego 14, 10-719 Olsztyn, Poland; 2 Department of Clinical Physiology, Faculty of Veterinary Medicine, University of Warmia and Mazury, Olsztyn, Poland; 3 Department of Animal Reproduction with Clinic, Faculty of Veterinary Medicine, University of Warmia and Mazury, Olsztyn, Poland; 4 Department of Pathophysiology, School of Medicine, University of Warmia and Mazury, Olsztyn, Poland

**Keywords:** Bisphenol a, Cholinergic innervation, Neuropeptides, Uterus

## Abstract

Bisphenol A, a substance commonly used in plastic manufacturing, is relatively well known as an endocrine disruptor, which may bind to estrogen receptors and has multidirectional negative effects on both human and animal organisms. Previous studies have reported that BPA may act on the reproductive organs, but knowledge concerning BPA-induced changes within the nerves located in the uterine wall is extremely scant. The aim of this study was to investigate the impact of various doses of BPA on the parasympathetic nerves located in the corpus and horns of the uterus using a single and double immunofluorescence method. The obtained results have shown that BPA may change not only the expression of vesicular acetylcholine transporter (VAChT—a marker of parasympathetic nervous structures) in the uterine intramural nerve fibers, but also the degree of colocalization of this substance with other neuronal factors, including substance P (SP), vasoactive intestinal polypeptide (VIP), galanin (GAL), and calcitonin gene–related peptide (CGRP). Moreover, BPA caused changes in the density of the overall populations of fibers immunoreactive to the particular neuropeptides mentioned above. The characteristics of the changes clearly depended on the part of the uterus, the neuronal factors studied, and the dosage of BPA. The mechanisms of the observed fluctuations are probably connected with the neurotoxic and/or pro-inflammatory activity of BPA. Moreover, the results have shown that even low doses of BPA are not neutral to living organisms. Changes in the neurochemical characterization of nerves supplying the uterine wall may be the first subclinical sign of intoxication with this substance.

## Introduction

Bisphenol A (BPA) is an organic substance, which is commonly used in the production of plastics (Gatimel et al. 2016). It is present in many everyday objects, including paints, bottles, containers, thermal paper, household products, and many other things (Mikołajewska et al. 2015). With regard to its chemical structure, BPA is similar to estrogen, resulting in the possibility of it having an impact on estrogen receptors (Zhang et al. 2018). This possibility makes this substance possibly have an adverse effect on living organisms and is classified as an endocrine disruptor. The particular danger of intoxication with BPA is connected with the use of this substance in plastics that have contact with food, including food containers, bottles, and the inner surface of tins, as well as plates and cutlery. BPA may pass into the food and water and get into the organism by the digestive system. Previous studies have shown that BPA has multidirectional negative effects on both human and animal organisms. Namely, it is known that BPA may affect immunological and endocrine systems, kidneys, the intestines, and the heart (Gramec Skledar and Peterlin Mašič 2016; Hu et al. 2016a, b, Rochester 2013) and exposure to this substance may result in an increased risk of diabetes, heart attack, hypertension, or cancer (Gao and Wang 2014; Seachrist et al. 2016). However, the most visible effects of BPA have been observed in the nervous and reproductive systems. The neurotoxic activity of BPA consists of disorders in the development of processes of neuronal cells, the inhibition of the extension of them, and changes in synaptogenesis (Xu et al. 2013). The mechanisms of these processes are not fully explained, but they are probably connected with BPA-induced changes in the intraneuronal calcium concentration (Feiteiro et al. 2018) as well as the synthesis and expression of neuronal active proteins, which have been observed both in the central and in the peripheral nervous systems (Szymanska et al. 2018). The changes mentioned above lead to disturbances in higher neuronal functions, such as behavior, memory, and/or learning (Johnson et al. 2016). Some neuronal symptoms may appear only after many months of exposure to BPA (Hutter et al. 2016), and in the light of the previous studies, the participation of BPA in the pathogenesis of neurodegenerative diseases, including Alzheimer’s or Parkinson’s diseases, cannot be excluded (Landolfi et al. 2017; Wang et al. 2017).

In turn, within the reproductive system, BPA causes changes in the uterine mucosal layer and endometrial stromal fibroblasts (Aghajanova and Giudice 2011). The exposure to BPA results in an increase in the uterine mucosa thickness with the simultaneous inhibition of apoptosis and the expression of estrogen receptors within this part of the uterine wall (Olson et al. 2017). In light of the previous studies, it is known that BPA may impair the estrus cycle and puberty (Nah et al. 2011), as well as disrupt embryo implantation and pregnancy. Even short exposure of the pregnant female to BPA may cause impaired development and anomalies in offspring and the first symptoms of these anomalies may occur only in the long term, during the adult life of the offspring (Susiarjo et al. 2015). Moreover, BPA can contribute to increase the following disease states in the reproductive system: atypical hyperplasia, stromal polyps, endometriosis, and cervical cancer (Buck Louis et al. 2013; Ma et al. 2015).

It should be underlined that information about the influence of BPA on the uterine nervous system is extremely scant (Rytel 2018), and studies concerning the effects of this substance on parasympathetic nervous structures located in the uterine wall do not even exist. On the other hand, it is commonly known that the parasympathetic nervous system plays very important roles in the regulation of the uterine physiological functions. Parasympathetic nerves in the uterine wall are processes of neurons located mainly in the inferior hypogastric ganglion and paracervical ganglion. The crucial neuromediator for this part of the nervous system is acetylcholine. It is known that acetylcholine may act on specific membrane receptors, such as five types of muscarinic (M_1–5_) and nicotinic receptors (Hedrick and Waters 2015), and in the uterine wall only muscarinic receptors M2 and M3 have been described (Yasuda et al. 2014). The main functions of acetylcholine in the uterus consist in stimulating the effects of muscles. Previous studies have shown that acetylcholine causes an increase in the frequency and intensity of uterine muscle contraction without significant fluctuations in the tone (Shu et al. 2017). Moreover, acetylcholine affects intrauterine blood vessels resulting in their dilation (Lamireau et al. 2002), as well as stimulating the secretory activity of uterine mucosa (Paris et al. 2000).

Of course acetylcholine is not the only neuronal active substance in the parasympathetic nervous system. It may colocalize with a wide range of other neuronal factors, which often play very different (and often opposite) functions. In light of the previous studies, it is known that all substances included in the present investigation, namely vasoactive intestinal polypeptide (VIP), substance P (SP), calcitonin gene–related peptide (CGRP), and galanin (GAL), may occur in the cholinergic nervous structures of various parts of the nervous system (Landis and Fredieu 1986; Lindeström and Ekblad 2002; Burnstock 2013). These substances probably play roles as neuromodulators, because their influence on cholinergic transmission has been reported. For example, GAL inhibits cholinergic neurotransmission in the heart (Potter and Smith-White 2005); SP increases the responsiveness of intestinal muscles to acetylcholine (Li et al. 2014), modulates cholinergic neurotransmission within intra-cardiac ganglia (Zhang et al.., Zhang et al. 2005), and together with CGRP modulates the function of nicotinic receptors on autonomic neurons (Di Angelantonio et al. 2003). Meanwhile, VIP enhances both the postjunctional effect of acetylcholine and the release of this mediator from nerve varicosities (Burnstock 2013).

It should be underlined that parasympathetic innervation also plays important protective and adaptive roles in pathological states of the uterus (Brauer 2017), which consist in the protection of neuronal and muscular tissues and homeostatic maintenance in conditions modified by pathogens or toxins. The most visible changes concern the neurochemical characterization of nervous structures, which may be the first subclinical symptoms of pathological states. Until now, such changes in parasympathetic innervation of the uterus have been observed during high exposure to hormones, inflammatory processes, and cancer (Collins et al. 2000; Possover et al. 2000). Previous studies conducted on the parasympathetic innervation of the liver and intestines (Thoene et al. 2018a; Szymanska and Gonkowski 2018) may also suggest that BPA may have an influence on this part of the nervous system. So the aim of the present study was to investigate for the first time the impact of low and high doses of BPA on parasympathetic nervous structures located in different parts of the porcine uterus, which might contribute to a better understanding of the mechanisms connected with BPA intoxication. Moreover, due to the relatively well-known similarities in neurochemical, physiological, and electrophysiological characteristics between the human and porcine nervous systems (Pohl et al. 2015), the obtained results may be the first step in determining an animal model of BPA-induced changes in the uterine innervation of humans.

## Materials and Methods

The present study was performed using 15 female immature pigs (Piétrain × Duroc breed, 18–20-kg body weight, at the age of about 8 weeks). All animals were kept in standard laboratory conditions with free access to water in the animal housing of the Faculty of Veterinary Medicine (University of Warmia and Mazury, Olsztyn, Poland). Sows were kept in pens (6 m^2^; five animals in each) and fed with the commercial feed “WIGOR 3” (WIPASZ S.A, Olsztyn, Poland), which was administered twice a day in daily portion as recommended by the manufacturer (1 kg/animal/day). All procedures performed during the study received the approval of the Local Ethical Committee responsible for experimental animals in Olsztyn (Poland) (decision numbers 28/2013 of 22 May 2013 and 65/2013/DLZ of 27 Nov 2013).

After an adaptation period, sows were divided into three groups (5 animals in each) according to the methods described previously by Szymanska et al. (2018). Within a short time, control sows (C group) received empty capsules once daily before the morning feeding (gelatin capsules, Carlson Laboratories, Arlington Heights, IL, USA), the animals from experimental I group (Ex I group) were treated with capsules with BPA (BISPHENOL A, 99 +%, product number: 239658-250G, Sigma-Aldrich, Poznan, Poland) at a dose of 0.05 mg/kg body weight (b.w.)/day, and the pigs from experimental II group (Ex II group) received capsules with BPA at a dose ten times higher (0.5 mg/kg b.w./day). Both animals from Ex I and Ex II groups received capsules in the same manner as the sows from C group. Every 5 days, all pigs were weighed to correlate the exact dose of BPA.

After 28 days of BPA administration, all pigs were euthanized. To this aim, the animals were premedicated with Stresnil (Janssen, Belgium, 75 μl/kg of b.w., given intramuscularly) and, after 20 min, an overdose of sodium thiopental (Thiopental, Sandoz, Kundl, Austria) was given intravenously.

Immediately after euthanasia, the uteri from all animals were collected. The organs were put into 4% buffered paraformaldehyde (pH 7.4) for 1 h (room temperature—rt) in order for fixation to occur. Then tissues were rinsed in phosphate buffer for 72 h, put into 18% phosphate-buffered sucrose and stored at 4 °C. After at least 3 weeks, the fragments of the uteri were included for further study, i.e., the fragment of the uterine corpus (c.0.5 cm long) located just below the uterine bifurcation and fragments of the left and right uterine horns (c. 0.5 cm long, positioned about 1 cm from the oviduct) was collected and frozen at − 22 °C. Then tissues were cut into 14-μm-thick sections with the cryostat (HM 525, Microm International, Germany) and mounted on microscopic slides. Slides were stored at − 20 °C until the single and double immunofluorescence labeling. This labeling was performed according to a previously described method (Majewski et al. 2002) using antibodies against vesicular acetylcholine transporter (VAChT—used here as a marker of parasympathetic nervous structures), substance P (SP), vasoactive intestinal polypeptide (VIP), galanin (GAL), and calcitonin gene–related peptide (CGRP) (the exact specification of antibodies is presented in Table 1). Slides with fragments of the uterus were dried for 1 h (rt) and blocked with solution with the following composition: 10% normal goat serum, 0.1% bovine serum albumin, 0.01% NaN3, 0.25% Triton x-100, and 0.05% thimerosal in PBS for 1 h in a humidity chamber (rt). Then slides with tissues were incubated overnight with one (single immunofluorescence technique) or a mixture of two (double immunofluorescence technique) antibodies received from different mammal species (humid chamber, rt). The next day, fragments of the uterus were incubated in the humidity chamber for 1 h (rt) with species specific secondary antibodies marked with various fluorochromes (Table 1). Then slides with the fragments of the uterus were treated with buffered glycerol and covered with coverslips. Between each stage of the labeling, slides were rinsed in PBS for 30 min. During the present study, a typical test of the labeling specificity was performed, such as the pre-absorption of the antisera with an appropriate antigen, omission, and replacement tests.

**Table 1:** List of antisera and reagents used in immunohistochemical investigations

Primary antibodies				
Antigen	Code	Species	Working dilution	Supplier
CGRP	MAB317	Mouse	1:1000	Millipore, Temecula, CA, USA
SP	8450-0505	Rat	1:1000	BioRad Hercules, CA,USA
VIP	9535-0504	Mouse	1:2000	Biogenesis Ltd., Poole, GB
GAL	T-5036	Guinea pig	1:2000	Peninsula Labs, San Carlos, CA, USA
VAChT	H-V007	Rabbit	1:2000	Phoenix Pharmaceuticals, Inc., Burlingame, CA, USA
Secondary antibodies				
Reagents			Working dilution	Supplier
Alexa fluor 488 donkey anti-mouse IgG			1:1000	ThermoFisher Scientific, Waltham, MA, USA
Alexa fluor 488 donkey anti-rat IgG			1:1000	ThermoFisher Scientific,
Alexa fluor 488 donkey anti-guinea pig IgG			1:1000	ThermoFisher Scientific,
Alexa fluor 546 donkey anti-rabbit IgG			1:1000	ThermoFisher Scientific,

Labeled fragments of the uterus were evaluated with an Olympus BX51 microscope equipped with epi-fluorescence and appropriate filter sets. The density of VAChT-like immunoreactive (LI) nerves was determined by the average number of such fibers *per* microscopic observation field (0.1 mm^2^). VAChT-LI nerves were counted in five randomly selected microscopic observation fields in the mucosal and muscular layers of six sections of the uteri (30 observation fields of the mucosal and muscular layers of each part of the uterus from each animal were included into the experiment), located at least 100 μm apart (to prevent double counting of the same nerves). During the counting, no attention was paid to the length of the nerves. Long, medium, and short nerve fibers were included into the study and counted as one fiber. Moreover, the density of nerves immunoreactive to VAChT, SP, VIP, GAL, and CGRP as a percentage of the area of the field in animals of all groups was measured using the computer program ImageJ2 Free (ImageJ developers, 2009). This evaluation was conducted in 30 areas of the field (of an area of 0.325 mm^2^) for the mucosal and muscular layers of each animal.

In turn, to determine the neurochemical characterization of VAChT-LI nerves, at least 500 such fibers located in the mucosal and muscular layers of each part of the uterus studied were examined for the presence of each other substance. The degree of co-localization of VAChT with other neuronal factors was shown as a percentage of nerves immunoreactive to the particular substances with regard to the number of VAChT-LI nerves (such nerves were considered representing 100%). In this part of the experiment, at least ten sections (located at least 100 μm apart) were evaluated from the particular parts of the uterus from each animal in order to study the co-localization of VAChT along with each of the other neuronal factors studied. During this counting, in a similar way to the counting of the VAChT-LI nerves, no attention was paid to the length of nerves, in which the co-localization of substances studied was noted. The microphotographs were made using an XM 10 digital camera (Olympus, Japan). The obtained data were pooled and presented as a mean ± SEM. Statistical analysis was carried out using Student’s *t* test (Statistica 9.1, StatSoft, Inc., Cracow, Poland). The differences were considered statistically significant at *p* ≤ 0.05.

## Results

### The Influence of BPA on the Number of VAChT-LI Nerves

**Fig. 1:**
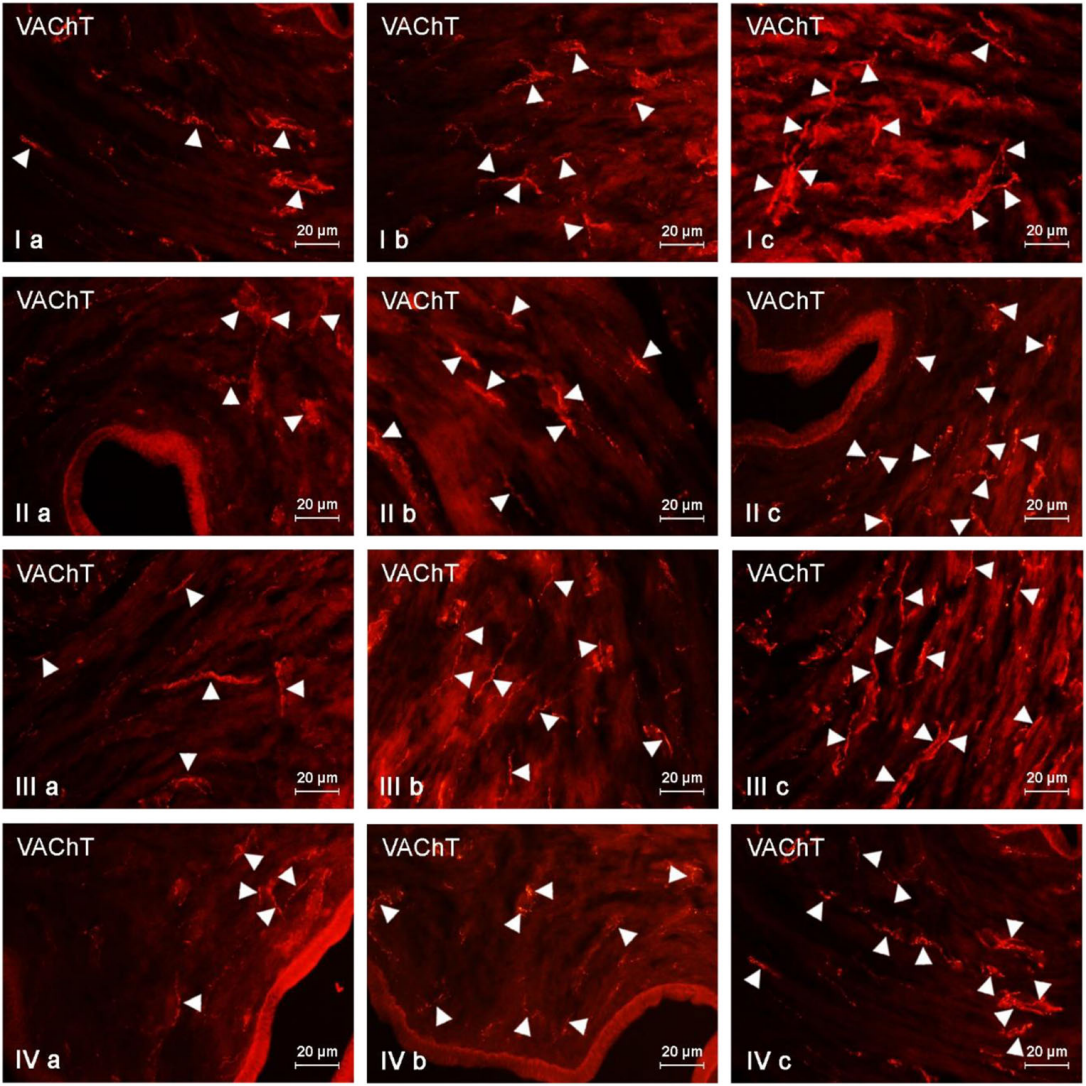
VAChT-positive nerves (VAChT+ green) in the (I) muscular layer of the uterine body, (II) mucosal layer of the uterine body, (III) muscular layer of the uterine horns, (IV) mucosal layer of the uterine horns of control animals (a) and pigs treated with low (b) and high (c) dose of bisphenol A. Nerves immunoreactive to VAChT are indicated with arroheads

During the present investigation, VAChT-positive nerves were observed in all parts of the uterus studied both under physiological conditions and after the impact of BPA (Fig. 1, Table 2, Table 3). In the muscular layer of the uterine corpus in control animals, the average number of such nerves amounted to above 27 fibers per observation field, while in the uterine horns these values oscillated around 20 fibers (Table 2). The number of VAChT-LI nerves in the uterine mucosa amounted to about 20 fibers per observation field, both in the uterine corpus and in the horns (Table 3). The influence of BPA on the number of VAChT-LI nerves in the wall of the uterus depended on the part of this organ, as well as the dose of the toxin. Low doses of BPA caused an increase in the number of VAChT-LI intramuscular nerves in the uterine corpus on average by about 7 fibers per observation field. An interesting situation was observed in the uterine horns. Namely, in the right horn, the administration of low doses of BPA resulted in an increase in the average number of VAChT-LI fibers (by around 5 fibers per observation field on average), while in the left horn the toxin did not affect the density of such nerves (Table 2). Simultaneously, the changes in the number of intramucosal VAChT-positive nerves under low doses of BPA were observed neither in the uterine corpus nor in the horns (Table 3).

**Table 2:** The number of VAChT-positive fibers and their neurochemical characterization in the muscular layer of the corpus and right and left horns of the uterus in control animals (C group) and pigs treated with low (E1 group) and high (E2 group) doses of bisphenol A

	Corpus of the uterus			Right horn			Left horn		
	C group	E1 group	E2 group	C group	E1 group	E2 group	C group	E1 group	E2 group
VAChT^+ 1)^	27.82 ± 2.48^ab^	34.25 ± 2.4^a^	36.75 ± 3.99^b^	21.66 ± 3.03^ab^	26.9 ± 3.46^a^	26.81 ± 4.6^b^	20.12 ± 1.96^b^	23.98 ± 4.24	29.47 ± 4.31^b^
VAChT^+^/SP^+ 2)^	12.39 ± 1.46^ab^	23.62 ± 1.13^a^	26.93 ± 1.51^b^	5.97 ± 0.97^ab^	11.56 ± 1.15^a^	12.25 ± 1.31^b^	6.29 ± 0.91^ab^	10.79 ± 0.96^ac^	13.2 ± 1.35^bc^
VAChT^+^/VIP^+ 2)^	24.46 ± 1.19^ab^	38.11 ± 3.54^ac^	51.81 ± 1.55^bc^	15.88 ± 2.11^ab^	32.33 ± 2.74^ac^	37.31 ± 2.23^bc^	16.36 ± 1.91^ab^	30.53 ± 2.15^ac^	39.86 ± 2.21^bc^
VAChT^+^/CGRP^+ 2)^	11.24 ± 0.88^ab^	20.47 ± 1.89^ac^	27.73 ± 1.42^bc^	6.74 ± 1.13^ab^	17.03 ± 1.18^ac^	20.46 ± 1.7^bc^	7.25 ± 0.79^ab^	16.24 ± 1.66^ac^	19.52 ± 1.62^bc^
VAChT^+^/GAL^+ 2)^	8.53 ± 0.85^ab^	11.55 ± 1.00^ac^	14.39 ± 1.57^bc^	6.39 ± 0.85^b^	8.02 ± 1.11	9.37 ± 0.94^b^	7.86 ± 1.52	6.13 ± 0.73	8.18 ± 1.47

^1)^The average number of fibers in the microscopic observation field (0.1 mm^2^)

^2)^The percentage of nerves immunoreactive to the particular substances with respect to all VAChT-positive nerves (VAChT-positive nerves were considered representing 100%)

^a^Differences for particular substances between C group and E1 group

^b^Differences for particular substances between C group and E2 group

^c^Differences for particular substances between E1 group and E2 group

The results were considered statistically significant *P* ≤ 0.05

**Table 3:** The number of VAChT-positive fibers and their neurochemical characterization in the mucosal layer of the corpus and right and left horns of the uterus in the control animals (C group) and pigs treated with low (E1 group) and high (E2 group) doses of bisphenol A

	Corpus of the uterus			Right horn			Left horn		
	C group	E1 group	E2 group	C group	E1 group	E2 group	C group	E1 group	E2 group
VAChT^+ 1)^	21.81 ± 2.92^b^	23.67 ± 3.82	27.15 ± 2.83^b^	18.82 ± 3.28^b^	18.25 ± 2.65	26.54 ± 3.58^b^	19.23 ± 3.64	18.79 ± 3.05	22.66 ± 2.68
VAChT^+^/SP^+ 2)^	3.59 ± 0.64^ab^	6.1 ± 0.93^a^	6.59 ± 1.19^b^	2.71 ± 0.51^ab^	4.03 ± 0.92^a^	4.58 ± 0.89^b^	3.07 ± 0.76^b^	3.71 ± 0.54^c^	4.86 ± 0.87^bc^
VAChT^+^/VIP^+ 2)^	12.41 ± 1.23^ab^	20.28 ± 2.05^bc^	32.8 ± 1.62^ac^	8.32 ± 1.21^b^	9.57 ± 1.46^c^	17.87 ± 1.76^bc^	7.41 ± 1.13^b^	10.33 ± 1.5^c^	18.76 ± 1.79^bc^
VAChT^+^/CGRP^+ 2)^	5.49 ± 0.88^ab^	15.63 ± 1.14^bc^	22.65 ± 2.22^bc^	3.74 ± 0.92^ab^	13.44 ± 0.98^ac^	18.18 ± 1.63^bc^	3.31 ± 0.87^ab^	13.65 ± 1.38^ac^	19.25 ± 2.12^bc^
VAChT^+^/GAL^+ 2)^	4.58 ± 0.72^ab^	6.15 ± 1.06^a^	7.14 ± 1.31^b^	3.86 ± 0.91b	4.11 ± 0.93	5.66 ± 0.74^b^	4.54 ± 0.94b	3.74 ± 0.79^c^	6.02 ± 0.86^bc^

^1)^The average number of fibers in the microscopic observation field (0.1 mm^2^)

^2)^The percentage of nerves immunoreactive to the particular substances with respect to all VAChT-positive nerves (VAChT-positive nerves were considered representing 100%)

^a^Differences for particular substances between C group and E1 group

^b^Differences for particular substances between C group and E2 group

^c^Differences for particular substances between E1 group and E2 group

The results were considered statistically significant *P* ≤ 0.05

High doses of BPA caused a clear increase (compared to control animals) in the number of intramuscular VAChT-LI nerves in the uterine corpus and horns (Table 2). These doses of BPA (contrary to low doses of this toxin) caused an increase in the average number of intramucosal VAChT-LI nerves in the uterine corpus (Table 3). Furthermore, the high doses of BPA caused an increase in the number of VAChT-LI nerves in the mucosal layer of the right horn, without any such changes in the nerves of the left horn (Table 3).

Both the character and severity of changes in the density of VAChT-LI nerves under the impact of low and high doses of BPA designated using the computer program ImageJ2 Free were compatible with the results obtained during the counting of the fibers in microscopic observation fields, presented above (Table 4).

**Table 4:** The density of nerve fibers immunoreactive to the particular substances in the muscular and mucosal layers of the corpus and right and left horns of the uterus in control animals (C group) and pigs treated with low (E1 group) and high (E2 group) doses of bisphenol A

	Corpus of the uterus			Right horn			Left horn		
	C group	E1 group	E2 group	C group	E1 group	E2 group	C group	E1 group	E2 group
Muscular layer									
VAChT	3.08 ± 0.32^ab^	4.12 ± 0.1^a^	4.28 ± 0.13^b^	3.01 ± 0.12^ab^	3.21 ± 0.08^a^	3.55 ± 0.07^b^	2.89 ± 0.09^b^	3.08 ± 0.09	3.44 ± 0.07^b^
SP	1.57 ± 0.04^ab^	1.86 ± 0.09^ac^	2.72 ± 0.11^bc^	1.08 ± 0.08^ab^	1.46 ± 0.08^ac^	2.13 ± 0.13^bc^	1.03 ± 0.07^ab^	1.38 ± 0.09^ac^	2.21 ± 0.12^bc^
VIP	2.35 ± 0.08^ab^	3.12 ± 0.09^bc^	4.11 ± 0.07^bc^	1.94 ± 0.11^ab^	2.74 ± 0.07^a^	2.88 ± 0.09^b^	2.00 ± 0.11^ab^	2.67 ± 0.06^a^	2.80 ± 0.08^b^
CGRP	1.97 ± 0.1^ab^	3.06 ± 0.09^ac^	3.62 ± 0.08^bc^	1.26 ± 0.07^ab^	1.72 ± 0.23^ac^	2.58 ± 0.19^bc^	1.31 ± 0.07^ab^	1.87 ± 0.06^ac^	2.61 ± 0.12^bc^
GAL	0.42 ± 0.07^ab^	0.69 ± 0.05^a^	0.76 ± 0.07^b+^	0.30 ± 0.05^ab^	0.44 ± 0.03^ac^	0.62 ± 0.29^bc^	0.24 ± 0.03^ab^	0.50 ± 0.04^a^	0.57 ± 0.05^b^
Mucosal layer									
VAChT	1.99 ± 0.15^b^	2.05 ± 0.11	2.29 ± 0.10^b^	1.46 ± 0.06^b^	1.69 ± 0.07	2.05 ± 0.05^b^	1.53 ± 0.07	1.75 ± 0.05	2.13 ± 0.06
SP	3.61 ± 0.09^ab^	4.62 ± 0.17^ac^	6.37 ± 0.14^bc^	2.07 ± 0.10^ab^	2.49 ± 0.13^ac^	4.54 ± 1.13^bc^	2.13 ± 0.09^ab^	2.68 ± 0.11^ac^	4.63 ± 0.14^bc^
VIP	1.73 ± 0.08^ab^	2.95 ± 0.19^ac^	4.13 ± 0.27^bc^	1.26 ± 0.17^ab^	1.68 ± 0.09^ac^	2.32 ± 0.07^bc^	1.15 ± 1.14^ab^	1.58 ± 0.09^ac^	2.21 ± 0.07^bc^
CGRP	1.25 ± 0.08^ab^	1.84 ± 0.07^ac^	2.59 ± 0.07^bc^	1.1 ± 0.08^ab^	1.85 ± 0.07^ac^	2.10 ± 0.16^bc^	1.17 ± 0.08^ab^	1.92 ± 0.08^a^	2.04 ± 0.11^b^
GAL	0.37 ± 0.05^b^	0.42 ± 0.08^c^	0.64 ± 0.06^bc^	0.22 ± 0.04^ab^	0.42 ± 0.04^a^	0.43 ± 0.04^b^	0.25 ± 0.05^ab^	0.47 ± 0.04^a^	0.39 ± 0.04^b^

The density of nerves are presented as an average percentage of the area of the field using the computer program ImageJ2 Free (ImageJ Developers, 2009)

^aD^ifferences for particular substances between C group and E1 group

^b^Differences for particular substances between C group and E2 group

^c^Differences for particular substances between E1 group and E2 group

The results were considered statistically significant *P* ≤ 0.05

### The Influence of BPA on the Neurochemical Characterization of VAChT-LI Nerves in the Muscular Layer of the Uterine Corpus

**Fig. 2:**
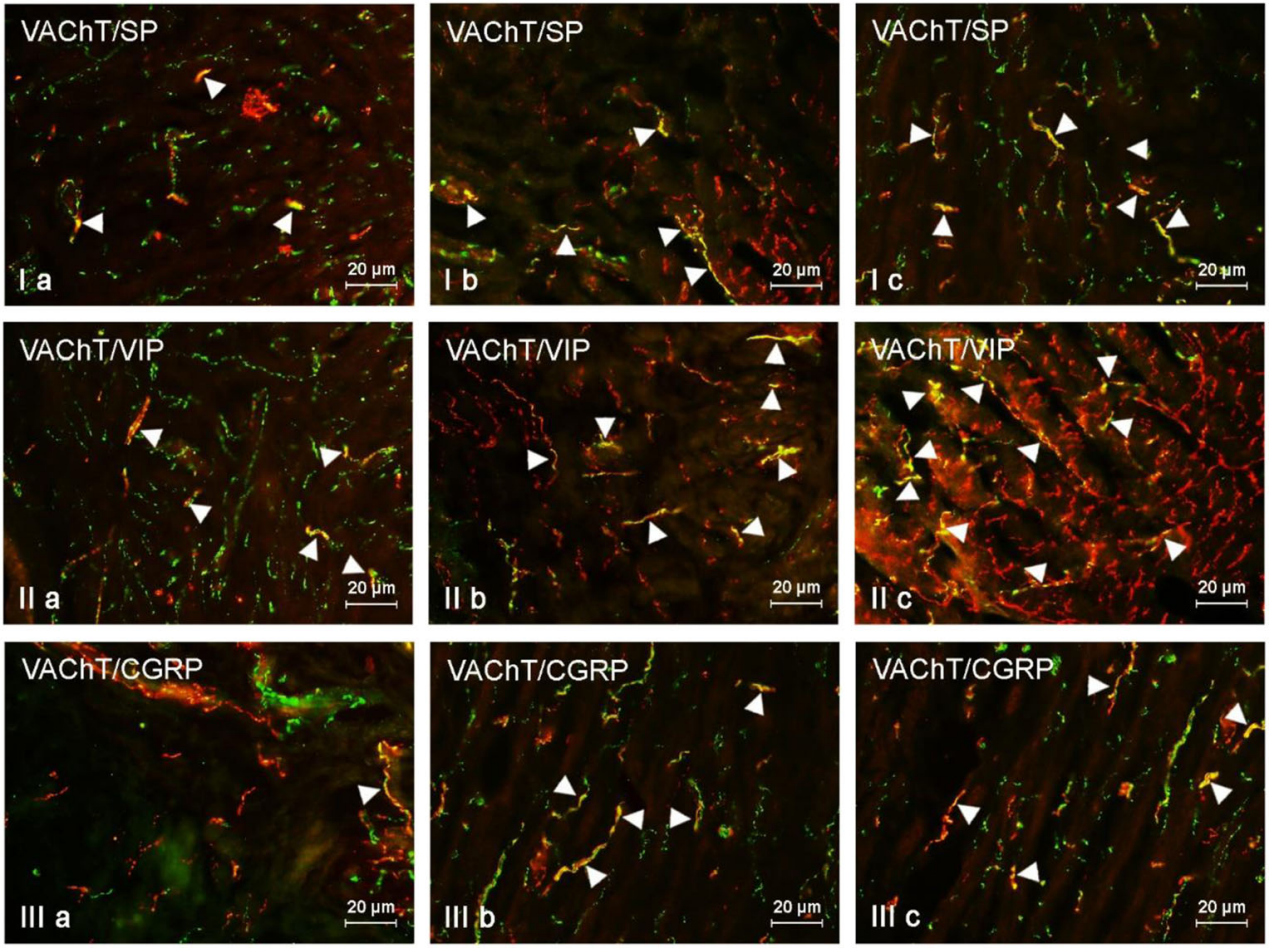
VAChT-positive nerves (VAChT+ red) immunoeractive to SP (I), VIP (II), and CGRP (III) (green) in the muscular layer of the uterine body of control animals (a) and pigs treated with low (b) and high (c) dose of bisphenol A. Nerves simultaneously immunoreactive to VAChT and other substances (yellow) are indicated with arrowheads

In the muscular layer of the uterine corpus under physiological conditions, the largest percentage of VAChT-positive nerves (more than 20% of all cholinergic fibers) were simultaneously immunoreactive to VIP (Fig. 2, Table 2). A slightly less number of cholinergic nerves showed the presence of SP and/or CGRP. In turn, GAL was observed in the least number of cholinergic nerves. Both low and high doses of BPA affected the neurochemical characterization of cholinergic nerves in the muscular layer of the uterine corpus (Table 2). The most visible changes concerned the number of fibers simultaneously immunoreactive to VAChT and VIP, the percentage of which was higher in comparison to the control animals by about 14 percentage points (pp) in the E1 group and by about 27 pp in the E2 group (Table 2).

BPA-induced changes were also found in VAChT+/SP+ and/or VAChT+/CGRP+ nerves (Fig. 2, Table 2). The percentage of the former increased by about 11 pp under low doses of BPA and about 14 pp in animals treated with high doses of the toxin. In the case of the latter (VAChT+/CGRP+ nerves), these values amounted to 9 pp and approximately 16 pp, respectively. The least visible changes were observed in VAChT+/GAL+ nerves. Their number was higher by about 3 pp in animals from the E1 group and about 6 pp in pigs from the E2 group.

### The Influence of BPA on the Neurochemical Characterization of VAChT-LI Nerves in the Muscular Layer of the Uterine Horns

**Fig. 3:**
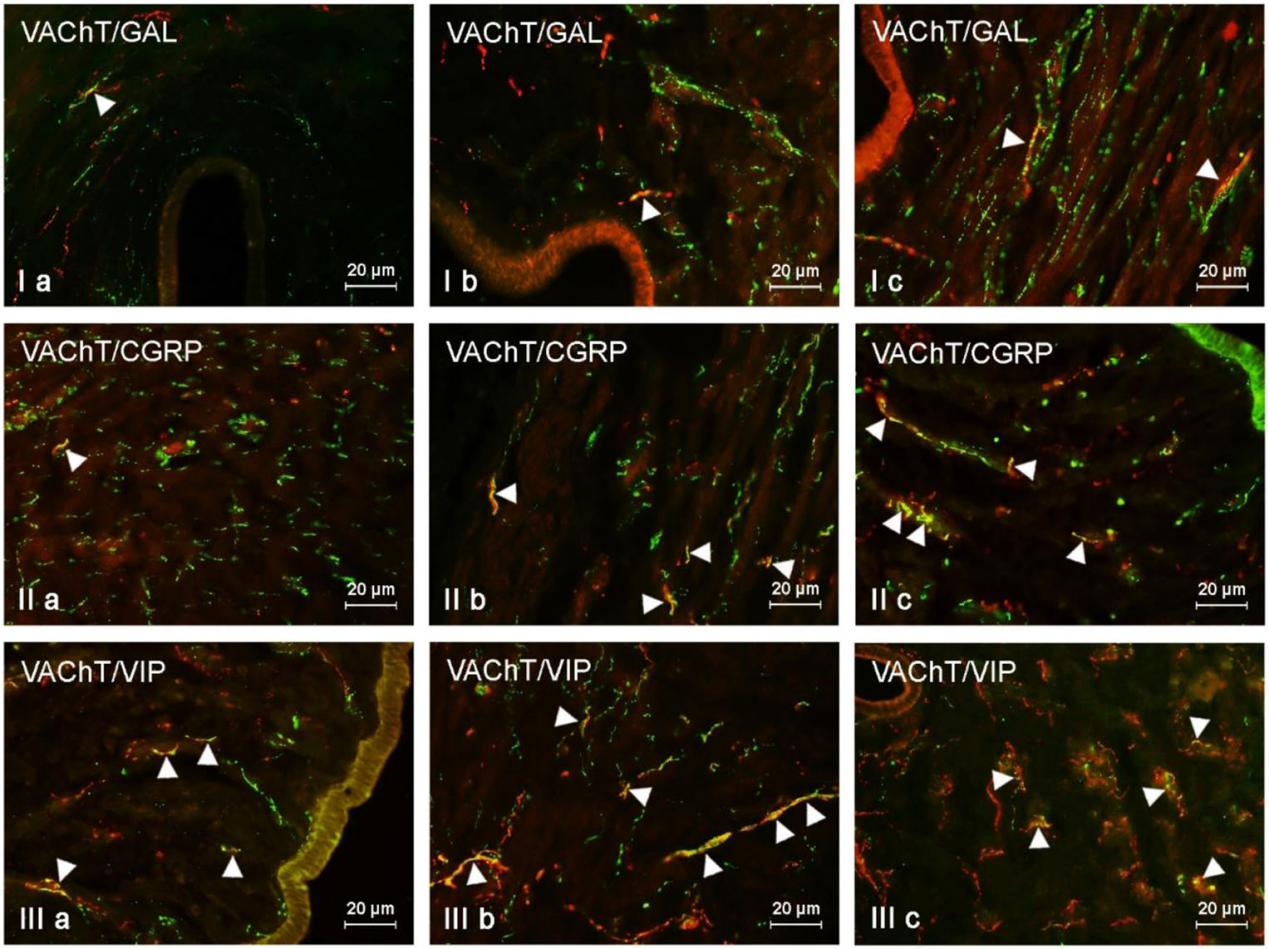
VAChT-positive nerves (VAChT+ red) immunoreactive to GAL (I), CGRP (II), and VIP (III) (green) in the mucosal layer of the uterine body of control animals (a) and pigs treated with low (b) and high (c) dose of bisphenol A. Nerves simultaneously immunoreactive to VAChT and other substances (yellow) are indicated with arrowheads

Under physiological conditions, the largest number of VAChT-LI nerves (slightly above 15% of all nerves immunoreactive to VAChT) located in the muscular layer of the uterine horns also contained VIP. The other neuronal factors studied were present at less than 10% of all nerves immunoreactive to VAChT (Fig. 3, Table 2). Low doses of BPA caused a clear increase in the number of VAChT+/VIP+ and/or VAChT+/CGRP+ nerves. The percentage of the former was higher in comparison to control animals by more than 14 pp, and the latter by above 9 pp. The administration of low doses of BPA also resulted in an increase in the number of VAChT-positive nerves showing the presence of SP (by above 4 pp). Contrary to other substances, low doses of BPA did not change the degree of co-localization of VAChT with GAL. In animals treated with high doses of BPA, the increase in the degree of co-localization of VAChT with the majority of other substances studied in comparison to the control group was observed, and the most visible changes concerned VAChT+/VIP+ fibers (an increase of more than 21 pp). In a similar way to low doses of BPA, high doses of the toxin did not affect the number of VAChT+/GAL+ nerves in the left horn, but interestingly increased the number of such fibers in the right horn (Fig. 3, Table 2).

### The Influence of BPA on the Neurochemical Characterization of VAChT-LI Nerves in the Mucosal Layer of the Uterine Corpus

**Fig. 4:**
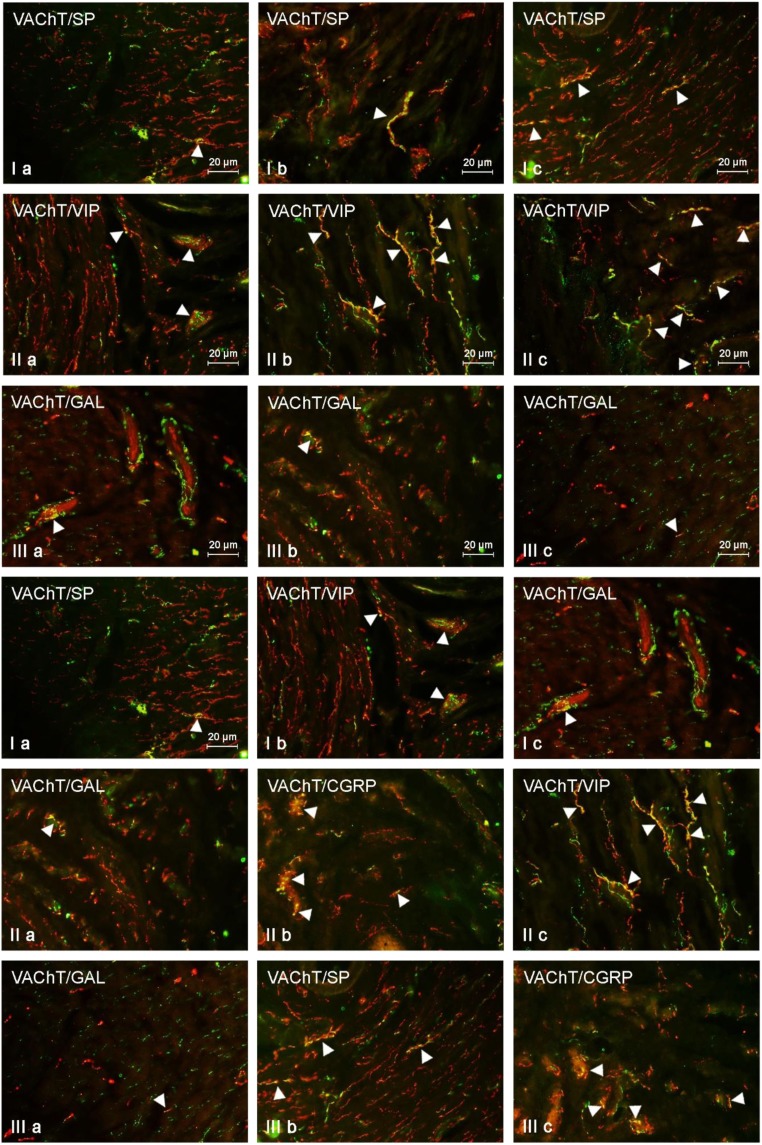
VAChT-positive nerves (VAChT+ red) immunoeractive to SP (I), VIP (II), and GAL (III) (green) in the muscular layer of the uterine horns of control animals (a) and pigs treated with low (b) and high (c) dose of bisphenol A. Nerves simultaneously immunoreactive to VAChT and other substances (yellow) are indicated with arrowheads

Under physiological conditions, the largest number of cholinergic nerves located in the mucosa of the uterine corpus also showed the presence of VIP. This peptide was observed in more than 12% of all VAChT-LI nerves (Fig. 4, Table 3). A clearly lower number of cholinergic nerves (below 6%) were simultaneously immunoreactive to CGRP, GAL, and/or SP (Fig. 4, Table 3).

The most visible changes induced by low doses of BPA concerned VAChT+/VIP+ and VAChT+/CGRP+ nerves. The increase in the former (in comparison to control pigs) was about 8 pp, while the latter increased by about 10 pp (Table 3). The increase of the percentage of VAChT+/SP+ and/or VAChT+/GAL+ nerves was significantly lower and did not exceed 3 pp (Table 3).

High doses of BPA caused more visible changes in the case of VAChT+/VIP+ and VAChT+/CGRP+ nerves (Fig. 4, Table 3). The percentage of VAChT+/VIP+ nerves in animals of the E2 group was higher in comparison to that in the control pigs by about 20 pp. In the case of VAChT+/CGRP+ fibers, this value amounted to 17 pp. The changes in the percentage of VAChT+/GAL+ and/or VAChT+/SP+ were less visible and achieved only about 3 pp (Table 3).

### The Influence of BPA on the Neurochemical Characterization of VAChT-LI Nerves in the Mucosal Layer of the Uterine Horns

In control animals, the largest number of VAChT-LI nerves located in the mucosal layer of the uterine horns showed the presence of VIP. This substance was noted in about 8% of all cholinergic nerves. Other substances studied were present in smaller numbers of VAChT-positive fibers, and the degree of co-localization of VAChT with other neuronal factors did not exceed 4% of all VAChT-positive nerves (Table 3).

**Fig. 5:**
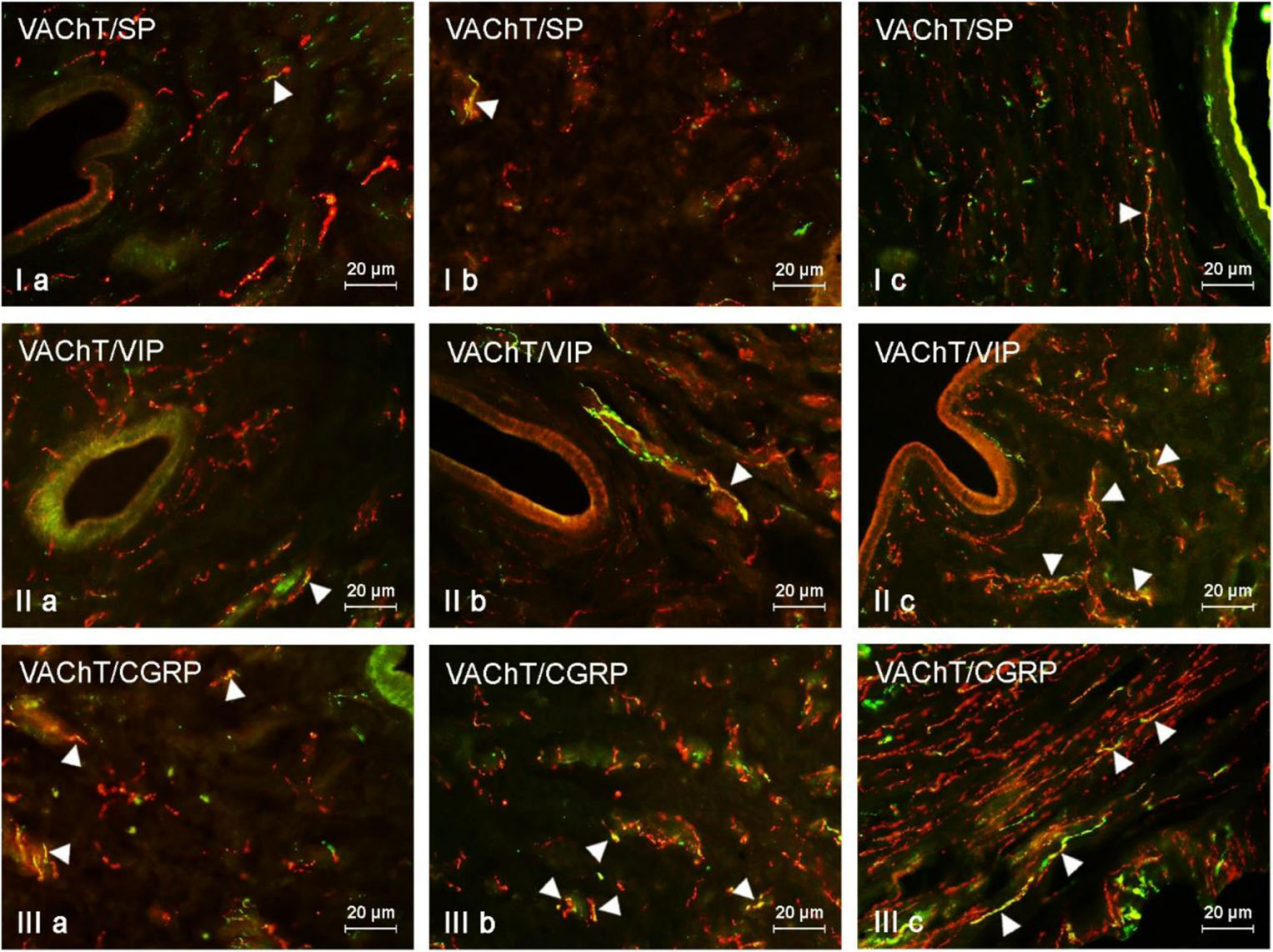
VAChT-positive nerves (VAChT+ red) immunoeractive to SP (I), VIP (II), and CGRP (III) (green) in the in the mucosal layer of the uterine horns of control animals (a) and pigs treated with low (b) and high (c) dose of bisphenol A. Nerves simultaneously immunoreactive to VAChT and other substances (yellow) are indicated with arrowheads

Low doses of BPA caused a clear increase (by about 10 pp in comparison to the control group) in the degree of co-localization of VAChT with CGRP (Table 3). Interestingly, the administration of low doses of BPA resulted in an increase in the percentage of VAChT+/SP+ nerves in the right horn (by about 2 pp), while it did not affect the same such nerves in the left horn. Moreover, low doses of BPA did not affect the number of VAChT+/VIP+ and/or VAChT+/GAL+ fibers (Fig. 5, Table 3).

In animals treated with high doses of BPA, the largest changes concerned VAChT+/CGRP+ and/or VAChT+/VIP+ fibers. The percentage of the former increased in comparison to that in the control pigs by about 15 pp, and the second by about 10 pp (Fig. 5, Table 3). Less visible changes (an increase of about 2 pp) were observed in the case of VAChT+/GAL+ and/or VAChT+/SP+ nerves (Fig. 5, Table 3).

### The Influence of BPA on Nerves Immunoreactive to VIP, SP, CGRP, and/or GAL

During the present study, it was demonstrated that both low and high doses of BPA not only change the density and neurochemical characterization of VAChT-LI nerves, but also result in fluctuations of the overall populations of fibers immunoreactive to the particular neuropeptides included in this study (Table 4).

In the muscular layer of the uterine corpus and horns under physiological conditions, the highest density was noted in the case of nerves immunoreactive to VIP (Table 4). In turn, the population of GAL-LI nerves was the lowest. Generally, both doses of BPA studied caused an increase in the density of nerves immunoreactive to the investigated neuropeptides, whereby the changes noted under the impact of the high doses of the toxin were much more visible in the animals treated with high doses of the toxin (Table 4). Moreover, clearer fluctuations were noted within the corpus of the uterus. Such a situation was observed in nerves immunoreactive to the majority of neuropeptides studied. The only exception was the population of nerves immunoreactive to GAL, the density of which was not modified by either low or high doses of BPA (Table 4).

In the mucosal layer of the control animals, the highest density was observed in the case of SP-LI nerves (Table 4). The least numerous population (similar to the muscular layer) was nerves immunoreactive to GAL (Table 4). Changes in the density of the intramucosal nerves immunoreactive to these particular neuropeptides were analogous to those noted within the muscular layer (Table 4).

## Discussion

Previous studies have shown that BPA—a substance commonly found in plastic articles—is dangerous for living organisms and excessive exposure to this substance may contribute to an increase in the incidence of various serious diseases and disturbances, including cancer, heart attack, diabetes, impairment of fertility, or abnormal changes in higher cortical functions (Gramec Skledar and Peterlin Mašič 2016; Hu et al. 2016a, b; Rochester 2013; Gao and Wang 2014; Seachrist et al. 2016). Despite the relatively well-known significant toxicity of BPA, many aspects connected with the effects of this substance on humans and animals are not fully clear. One of them is the level of tolerable daily intake (TDI) of BPA. Due to the fact that the lowest dose of BPA, at which symptoms of intoxication have been observed, clearly depends on the internal organ, the species of animal, the method of administration, and the duration of the exposure (Rochester 2013; Szymanska et al. 2018; Thoene et al. 2018), establishment of a safe dose for this substance is very difficult. For a long time according to European Food Safety Authority (EFSA), the TDI of BPA was established at a dose 0.05 mg/kg b.w./day (EFSA 2006). However, some studies have shown that this dose may affect the immune system (Halimi et al. 2016) and therefore in January 2015, the EFSA reduced the TDI of BPA to 4 μg/kg b.w./day (EFSA 2015). The latter dosage has been made temporary, and the determination of a definitive TDI for BPA depends on further toxicological studies.

The results obtained during the present investigation have shown that a dose of BPA at the level of 0.05 mg/kg b.w./day indeed causes changes in the expression of neuronal factors in the parasympathetic nerves supplying the uterine wall. Together with previous studies on the enteric nervous system (Szymanska et al. 2018), these observations may suggest that the abovementioned dosage is not neutral for living organisms, and changes in the nervous system may be the first subclinical signs of intoxication with BPA. In light of this data, the decision of the EFSA to reduce the TDI of BPA was fully justified.

It should be underlined that the exact mechanisms of the observed changes have not yet been fully explained. Fluctuations in the expression of neuromediators may be the result of the direct neurotoxic effects of BPA. Such effects have been reported in previous studies (Viberg and Lee 2012), where the influence of BPA on synapses and dendrites has been shown. Most likely, the changes observed in the present study arise from protective and adaptive processes. This thesis is supported by the fact that during the present investigation BPA-induced changes were noted in populations of nerves immunoreactive to almost all neuronal factors studied, and all these substances are known as neuroprotective factors. In the light of previous studies, among substances included into this experiment, VIP is the most powerful protective factor. This peptide increases the survivability of neurons undergoing damage from various agents, such as inflammation, intoxication, and axotomy (Rytel and Calka 2016a, b; Makowska et al. 2018). This protection is achieved by inducing stimulatory effects in glial cells (Li et al. 2018). The protective and adaptive functions of other substances studied are less known, but previous studies have reported that GAL preserves brain cells during neurodegenerative processes (Lang et al. 2015), SP protects neurons against apoptosis (Lallemend et al. 2003), and CGRP, which is first of all a sensory neuromediator and/or neuromodulator (Luger 2002), takes part in repair processes within the nervous tissue (Rytel and Całka 2016b; Makowska and Gonkowski 2018). It cannot be excluded that the increase of VAChT-LI nerves noted in the present study under the impact of BPA may be connected with an abnormal neurogenesis of this toxin. Until now, such neurogenesis has only been described within the central nervous system and it is known that this phenomenon results from a BPA-induced increase in the number and activity of glial cells leading to alterations in the expression of neuronal factors and changes in the neuro-inflammatory status of the nervous tissue (Takahashi et al. 2018). The mechanisms of BPA-induced neurogenesis in the peripheral nervous system are unknown, but probably it is closely linked to the pro-inflammatory activity of this toxin (Savastano et al. 2015), which is further described in a later section of this article.

The second possible mechanism of observed changes, especially in nerves located in the muscular layer, may be connected with the influence of BPA on the smooth muscles. Previous studies have reported that BPA causes relaxatory effects on the muscles within the digestive tract and uterus (Szymanska et al. 2018; Rytel 2018), but mechanisms of this action still remain not entirely clear. Some in vitro experiments on the uterus of rodents have shown that BPA inhibits the expression of contractile proteins taking part in oxytocin and prostaglandin-related pathways (An et al. 2013). Other studies have reported that BPA affects the expression of calmodulin and the phosphorylation of calcium/calmodulin dependent kinase in male reproductive organs (Qian et al. 2014), which suggests that the influence of this toxin concerns muscular contraction and is connected with disorders of calcium ion current. This thesis is supported by observations on the various parts of the nervous system, where BPA significantly changes the intracellular concentration of Ca^2+^. Moreover, processes connected with BPA-induced influence on the uterine muscles are probably similar to mechanisms taking place in the heart, in which these processes have been investigated in detail. Namely, in the heart, BPA inhibits electrical conduction (Posnack et al. 2014), influences protein kinase A (PKA) and Ca(2+)/CaM-dependent protein kinase II (CAMKII) signaling pathways (Gao et al. 2013), and also affects the handling of calcium ions (Ramadan et al. 2018). Moreover, it is known that BPA may affect neuronal nicotinic receptors (Borea et al. 2004). Given the above, BPA-induced fluctuations in the expression of VAChT as well as changes in the neurochemical characterization of cholinergic nerves observed during the present study may be the result of a direct impact of the toxin on muscles and neuromuscular junctions. Moreover, because the relaxatory effects of BPS have been reported in previous studies (Jones et al. 2016), it stands to reason that the increase in the number cholinergic nerves noted after BPA administration is rather due to a suppression of acetylcholine release than an increase in the synthesis of this neuromediator.

The changes observed during the present study may also arise from the relatively well-known pro-inflammatory characteristics of BPA. Previous studies have reported that this substance intensifies the synthesis of pro-inflammatory cytokines, such as TNF-α and IL-6 (Savastano et al. 2015) with a simultaneous decrease in the expression of anti-inflammatory factors, including IL-10 and transforming growth factor-β (TGF-β) (Hu et al. 2016a, b). It is also known that BPA increases IgG2 and IgA secretion and suppresses macrophage adhesion (Segura et al. 1999). The pro-inflammatory action of BPA also consists in the induction of prostaglandin-endoperoxide synthase 2 (COX 2) by the mitogen-activated protein kinase pathway (Song et al. 2017) and the suppression of lymphocyte mitogenesis (Sakazaki et al. 2002). On the other hand, it is commonly accepted that the nervous system plays a crucial role in the inflammatory processes, and fluctuation in the expression of neuromediators and/or neuromodulators is one of the first reactions of the nervous tissue to inflammation (Ackermann 2013). Moreover, all neuronal substances studied during the present investigation are factors that take part in inflammatory and immunological mechanisms. Namely, SP has pro-inflammatory properties that act on receptors localized to immune cells and increases TNF-α expression. VIP stimulates the production of IL-1α, IL-1β, IL-3, and IL-6 within glial cells (Choi et al. 2013). Moreover, this peptide inhibits macrophage activity (Delgado et al. 1999). In turn, GAL causes an increase in the expression of IFN-γ and IL-12/23 (Koller et al. 2017) with a simultaneous inhibition in the synthesis of TNF-α and IL-1β (Oztas et al. 2017). The roles of CGRP in inflammatory mechanisms are the most obscure. Nevertheless, it is known that the expression of this substance within the autonomic peripheral nervous system increases during inflammatory processes. Moreover, the latest research has shown that CGRP suppresses the expression of inflammatory cytokines in the macrophages and increases the expression of anti-inflammatory factors (Duan et al. 2017). So changes in the expression of neuronal factors may result from inflammatory processes. Admittedly, during the experiment, the symptoms of inflammation were not observed, but fluctuations in neuromediators and/or neuromodulators may be the first subclinical signs of pathological processes (Barbosa-Cobos et al. 2018), and such a situation may have taken place in the case of the present study.

Nevertheless, BPA-induced changes observed during the present study may also result from other processes. Namely, they may be connected with the modulation of conduction of sensory and pain stimuli. It is known that SP and CGRP are the crucial sensory factors occurring in neuronal cells (Bulut et al. 2008). Although their most important roles are not connected with sensory stimuli conduction, GAL and VIP have been reported in sensory neuronal structures (Rytel and Calka 2016a, b). Moreover, it is known that the expression of all the abovementioned substances in sensory neurons increases during pathological factors, including inflammatory processes, nerve damage, and/or intoxication (Rytel and Calka 2016a, b). However, the hypothesis that the changes observed in the present study are the result of sensory or pain stimuli is unlikely, because the doses of BPA used in the experiment were relatively low and should not cause pain symptoms. Moreover, such symptoms were not observed in the experimental animals. Even so, it cannot be excluded that changes in the neurochemical characterization noted in the experiment concern sensory nerves, because the previous studies have reported that such nerves not only participate in sensory and pain stimuli conduction, but also are involved in homeostasis maintenance and the initiation of repair processes after damage (Rytel and Calka 2016a, b).

It is difficult to determine the consequences of BPA-induced changes in cholinergic innervation for functional activity of the uterus. During the present study, no clinical signs of the administration of BPA were observed in the experimental animals. Moreover, there were no macroscopic changes in the corpus and horns of the uterus under the influence of both low and high doses of the toxin. Considering the fact that acetylcholine in the uterus causes the contraction of muscles, dilation of intramural vessels, and the stimulation of secretory activity (Paris et al. 2000; Lamireau et al. 2002; Shu et al. 2017), these kinds of effects are to be logically expected. On the other hand, previous studies have reported that BPA acts on the uterine muscles as a strong relaxant factor (Salleh et al. 2015). Considering this, it is very likely that the increase in the number of VAChT-positive nerves was not the result of the intensification of acetylcholine synthesis, but was connected with the BPA-induced blocking of acetylcholine release from the nerve endings and/or disorders in intraneuronal transport. Nevertheless, the determination of consequences from the observed changes for uterine activity requires further studies.

Moreover, the present investigation has shown that not all of the neuronal substances studied play equal roles in response to uterine innervation after BPA exposure. Namely the obtained observations have shown that BPA affects both the density of GAL-LI nerves and the degree of co-localization of VAChT with GAL in some small way, which may suggest that this neuropeptide, despite its neuroprotective and adaptive functions (Lang et al. 2015; Koller et al. 2017), is not the key factor in the mechanisms taking place in uterine innervation during BPA-induced intoxication.

In conclusion, the obtained results have clearly indicated that even relatively low doses of BPA administered for only a short time are not neutral to the living organism and may cause changes in the neurochemical characterization of parasympathetic nerves located in the uterine wall. These observations suggest that the decision of the EVSA concerning the reduction of the tolerable daily intake of BPA was correct. However, due to the multidirectional negative effects of BPA, as well as the various functions of neuromediators and/or neuromodulators included in this experiment, the exact elucidation of mechanisms underlying these observed changes has not been easy. Most likely, the fluctuations in the neurochemical characterization of the uterine nerves are connected with the neurotoxic and pro-inflammatory properties of BPA, but other reasons (such as the direct impact on muscles, changes in sensory stimuli conduction, or genotoxic effects of BPA) cannot be excluded. Therefore, an explanation that includes all aspects connected with the influence of BPA on uterine innervation requires further investigation.

## Abbreviations

BPA: Bisphenol A
CGRP: Calcitonin gene–related peptide
EFSA: European Food Safety Authority
GAL: Galanin
LI: Like immunoreactive
SP: Substance P
TDI: Tolerable daily intake
VAChT: Vesicular acetylcholine transporter
VIP: Vasoactive intestinal polypeptide

## References

[CR1] Ackermann PW (2013). Neuronal regulation of tendon homoeostasis. Int J Exp Pathol.

[CR2] Aghajanova L, Giudice LC (2011). Effect of bisphenol A on human endometrial stromal fibroblasts in vitro. Reprod BioMed Online.

[CR3] An BS, Ahn HJ, Kang HS, Jung EM, Yang H, Hong EJ, Jeung EB (2013). Effects of estrogen and estrogenic compounds, 4-tert-octylphenol, and bisphenol A on the uterine contraction and contraction-associated proteins in rats. Mol Cell Endocrinol.

[CR4] Barbosa-Cobos RE, Lugo-Zamudio G, Flores-Estrada J, Becerril-Mendoza LT, Rodríguez-Henríquez P, Torres-González R, Moreno-Eutimio MA, Ramirez-Bello J, Moreno J (2018). Serum substance P: an indicator of disease activity and subclinical inflammation in rheumatoid arthritis. Clin Rheumatol.

[CR5] Borea PA, Varani K, Gessi S, Merighi S, Dal Piaz A, Gilli P, Gilli G (2004). Receptor binding thermodynamics at the neuronal nicotinic receptor. Curr Top Med Chem.

[CR6] Brauer MM (2017). Plasticity in uterine innervation: state of the art. Curr Protein Pept Sci.

[CR7] Buck Louis GM, Peterson CM, Chen Z, Croughan M, Sundaram R, Stanford J, Varner MW, Kennedy A, Giudice L, Fujimoto VY, Sun L, Wang L, Guo Y, Kannan K (2013). Bisphenol A and phthalates and endometriosis: the endometriosis: natural history, diagnosis and outcomes study. Fertil Steril.

[CR8] Bulut K, Felderbauer P, Deters S, Hoeck K, Schmidt-Choudhury A, Schmidt WE, Hoffmann P (2008). Sensory neuropeptides and epithelial cell restitution: the relevance of SP- and CGRP-stimulated mast cells. Int J Color Dis.

[CR9] Burnstock G (2013). Cotransmission in the autonomic nervous system. Handb Clin Neurol.

[CR10] Choi BK, Jeong SH, Cho JH, Shin HS, Son SW, Yeo YK, Kang HG (2013). Effects of oral deoxynivalenol exposure on immune-related parameters in lymphoid organs and serum of mice vaccinated with porcine parvovirus vaccine. Mycotoxin Res.

[CR11] Collins JJ, Wilson K, Fischer-Colbrie R, Papka RE (2000). Distribution and origin of secretoneurin-immunoreactive nerves in the female rat uterus. Neuroscience.

[CR12] Delgado M, Sun W, Leceta J, Ganea D (1999). VIP and PACAP differentially regulate the costimulatory activity of resting and activated macrophages through the modulation of B7.1 and B72 expression. J Immunol.

[CR13] Di Angelantonio S, Giniatullin R, Costa V, Sokolova E, Nistri A (2003). Modulation of neuronal nicotinic receptor function by the neuropeptides CGRP and substance P on autonomic nerve cells. Br J Pharmacol.

[CR14] Duan JX, Zhou Y, Zhou AY, Guan XX, Liu T, Yang HH, Xie H, Chen P (2017). Calcitonin gene-related peptide exerts anti-inflammatory property through regulating murine macrophages polarization in vitro. Mol Immunol.

[CR15] Feiteiro J, Mariana M, Glória S, Cairrao E (2018). Inhibition of L-type calcium channels by bisphenol A in rat aorta smooth muscle. J Toxicol Sci.

[CR16] Gao X, Wang HS (2014). Impact of bisphenol a on the cardiovascular system - epidemiological and experimental evidence and molecular mechanisms. Int J Environ Res Public Health.

[CR17] Gao X, Liang Q, Chen Y, Wang HS (2013). Molecular mechanisms underlying the rapid arrhythmogenic action of bisphenol A in female rat hearts. Endocrinology.

[CR18] Gatimel N, Lacroix MZ, Chanthavisouk S, Picard-Hagen N, Gayrard V, Parinaud J, Léandri RD (2016). Bisphenol A in culture media and plastic consumables used for ART. Hum Reprod.

[CR19] Gramec Skledar D, Peterlin Mašič L (2016). Bisphenol A and its analogs: do their metabolites have endocrine activity?. Environ Toxicol Pharmacol.

[CR20] Halimi A, Benyahia H, Bahije L, Adli H, Azeroual MF, Zaoui F (2016). A systematic study of the release of bisphenol A by orthodontic materials and its biological effects. Int Orthod.

[CR21] Hedrick T, Waters J (2015). Acetylcholine excites neocortical pyramidal neurons via nicotinic receptors. J Neurophysiol.

[CR22] Hu J, Wang Y, Xiang X, Peng C, Gao R, Goswami R, Zhou H, Zhang Y, Zhen Q, Cheng Q, Yang S, Li Q (2016). Serum bisphenol A as a predictor of chronic kidney disease progression in primary hypertension: a 6-year prospective study. J Hypertens.

[CR23] Hu Y, Zhang L, Wu X, Hou L, Li Z, Ju J, Li Q, Qin W, Li J, Zhang Q, Zhou T, Zhang L, Xu C, Fang Z, Zhang Y (2016). Bisphenol A, an environmental estrogen-like toxic chemical, induces cardiac fibrosis by activating the ERK1/2 pathway. Toxicol Lett.

[CR24] Hutter HP, Kundi M, Hohenblum P, Scharf S, Shelton JF, Piegler K, Wallner P (2016). Life without plastic: A family experiment and biomonitoring study. Environ Res.

[CR25] Johnson SA, Javurek AB, Painter MS, Ellersieck MR, Welsh TH, Camacho L, Lewis SM, Vanlandingham MM, Ferguson SA, Rosenfeld CS (2016). Effects of developmental exposure to bisphenol A on spatial navigational learning and memory in rats: a CLARITY-BPA study. Horm Behav.

[CR26] Jones BA, Wagner LS, Watson NV (2016). The effects of bisphenol A exposure at different developmental time points in an androgen-sensitive neuromuscular system in male rats. Endocrinology.

[CR27] Koller A, Bianchini R, Schlager S, Münz C, Kofler B, Wiesmayr S (2017). The neuropeptide galanin modulates natural killer cell function. Neuropeptides.

[CR28] Lallemend F, Lefebvre PP, Hans G, Rigo JM, Van de Water TR, Moonen G, Malgrange B (2003). Substance P protects spiral ganglion neurons from apoptosis via PKC-Ca2+-MAPK/ERK pathways. J Neurochem.

[CR29] Lamireau D, Nuyt AM, Hou X, Bernier S, Beauchamp M, Gobeil F, Lahaie I, Varma DR, Chemtob S (2002). Altered vascular function in fetal programming of hypertension. Stroke.

[CR30] Landis SC, Fredieu JR (1986). Coexistence of calcitonin gene-related peptide and vasoactive intestinal peptide in cholinergic sympathetic innervation of rat sweat glands. Brain Res.

[CR31] Landolfi A, Troisi J, Savanelli MC, Vitale C, Barone P, Amboni M (2017). Bisphenol A glucuronidation in patients with Parkinson’s disease. Neurotoxicology.

[CR32] Lang R, Gundlach AL, Holmes FE, Hobson SA, Wynick D, Hökfelt T, Kofler B (2015). Physiology, signaling, and pharmacology of galanin peptides and receptors: three decades of emerging diversity. Pharmacol Rev.

[CR33] Li C, Micci MA, Murthy KS, Pasricha PJ (2014). Substance P is essential for maintaining gut muscle contractility: a novel role for coneurotransmission revealed by botulinum toxin. Am J Physiol Gastrointest Liver Physiol.

[CR34] Li Y, Ge Y, Zhu W, Gong J, Cao L, Guo Z, Gu L, Li J (2018). Increased enteric glial cells in proximal margin of resection is associated with postoperative recurrence of Crohn's disease. J Gastroenterol Hepatol.

[CR35] Lindeström LM, Ekblad E (2002). Origins and projections of nerve fibres in rat pyloric sphincter. Auton Neurosci.

[CR36] Luger TA (2002). Neuromediators--a crucial component of the skin immune system. J Dermatol Sci.

[CR37] Ma XF, Zhang J, Shuai HL, Guan BZ, Luo X, Yan RL (2015). IKKβ/NF-κB mediated the low doses of bisphenol A induced migration of cervical cancer cells. Arch Biochem Biophys.

[CR38] Majewski M, Kaleczyc J, Wasowicz K, Bossowska A, Gonkowski S, Klimaschewski L (2002). Characterization of afferent and efferent galanin-containing nerve fibres in the porcine ovary. Folia Histochem Cytobiol.

[CR39] Makowska K, Gonkowski S. The influence of inflammation and nerve damage on the neurochemical characterization of calcitonin gene-related peptide-like immunoreactive (CGRP-LI) neurons in the enteric nervous system of the porcine descending colon. Int J Mol Sci 2018, 19, pii: E548. doi: 10.3390/ijms19020548. 10.3390/ijms19020548 PMC5855770 29439512

[CR40] Makowska K, Obremski K, Gonkowski S (2018). The impact of T-2 toxin on vasoactive intestinal polypeptide-like Immunoreactive (VIP-LI) nerve structures in the wall of the porcine stomach and duodenum. Toxins (Basel).

[CR41] Mikołajewska K, Stragierowicz J, Gromadzińska J (2015). Bisphenol A - application, sources of exposure and potential risks in infants, children and pregnant women. Int J Occup Med Environ Health.

[CR42] Nah WH, Park MJ, Gye MC (2011). Effects of early prepubertal exposure to bisphenol A on the onset of puberty, ovarian weights, and estrous cycle in female mice. Clin Exp Reprod Med.

[CR43] Olson MR, Su R, Flaws JA, Fazleabas AT (2017). Bisphenol A impairs decidualization of human uterine stromal fibroblasts. Reprod Toxicol.

[CR44] Oztas B, Sahin D, Kir H, Eraldemir FC, Musul M, Kuskay S, Ates N (2017). The effect of leptin, ghrelin, and neuropeptide-Y on serum Tnf-Α, Il-1β, Il-6, Fgf-2, galanin levels and oxidative stress in an experimental generalized convulsive seizure model. Neuropeptides.

[CR45] Paris JM, Williams KJ, Hermsmeyer KR, Delansorne R (2000). Nomegestrol acetate and vascular reactivity: nonhuman primate experiments. Steroids.

[CR46] Pohl CS, Medland JE, Moeser AJ (2015). Early-life stress origins of gastrointestinal disease: animal models, intestinal pathophysiology, and translational implications. Am J Physiol Gastrointest Liver Physiol.

[CR47] Posnack NG, Jaimes R, Asfour H, Swift LM, Wengrowski AM, Sarvazyan N, Kay MW (2014). Bisphenol A exposure and cardiac electrical conduction in excised rat hearts. Environ Health Perspect.

[CR48] Possover M, Stöber S, Plaul K, Schneider A (2000). Identification and preservation of the motoric innervation of the bladder in radical hysterectomy type III. Gynecol Oncol.

[CR49] Potter EK, Smith-White MA (2005) Galanin modulates cholinergic neurotransmission in the heart. Neuropeptides 39(3):345–348 10.1016/j.npep.2004.12.006 15944033

[CR50] Qian W, Zhu J, Mao C, Liu J, Wang Y, Wang Q, Liu Y, Gao R, Xiao H, Wang J (2014). Involvement of CaM-CaMKII-ERK in bisphenol A-induced Sertoli cell apoptosis. Toxicology.

[CR51] Ramadan M, Sherman M, Jaimes R, Chaluvadi A, Swift L, Posnack NG (2018). Disruption of neonatal cardiomyocyte physiology following exposure to bisphenol-a. Sci Rep.

[CR52] Rochester JR (2013). Bisphenol A and human health: a review of the literature. Reprod Toxicol.

[CR53] Rytel L. The influence of bisphenol A (BPA) on neuregulin 1-like Immunoreactive nerve fibers in the wall of porcine uterus. Int J Mol Sci 2018, 19, pii: E2962. doi: 10.3390/ijms19102962. 10.3390/ijms19102962 PMC6213500 30274171

[CR54] Rytel L, Calka J (2016). Acetylsalicylic acid-induced changes in the chemical coding of extrinsic sensory neurons supplying the prepyloric area of the porcine stomach. Neurosci Lett.

[CR55] Rytel L, Calka J (2016). Neuropeptide profile changes in sensory neurones after partial prepyloric resection in pigs. Ann Anat.

[CR56] Sakazaki H, Ueno H, Nakamuro K (2002). Estrogen receptor alpha in mouse splenic lymphocytes: possible involvement in immunity. Toxicol Lett.

[CR57] Salleh N, Giribabu N, Feng AO, Myint K (2015). Bisphenol A, dichlorodiphenyltrichloroethane (DDT) and vinclozolin affect ex-vivo uterine contraction in rats via uterotonin (prostaglandin F2α, acetylcholine and oxytocin) related pathways. Int J Med Sci.

[CR58] Savastano S, Tarantino G, D'Esposito V, Passaretti F, Cabaro S, Liotti A, Liguoro D, Perruolo G, Ariemma F, Finelli C, Beguinot F, Formisano P, Valentino R (2015). Bisphenol-A plasma levels are related to inflammatory markers, visceral obesity and insulin-resistance: a cross-sectional study on adult male population. J Transl Med.

[CR59] Seachrist DD, Bonk KW, Ho SM, Prins GS, Soto AM, Keri RA (2016). A review of the carcinogenic potential of bisphenol A. Reprod Toxicol.

[CR60] Segura JJ, Jiménez-Rubio A, Pulgar R, Olea N, Guerrero JM, Calvo JR (1999). In vitro effect of the resin component bisphenol A on substrate adherence capacity of macrophages. J Endod.

[CR61] Shu SJ, Lei XG, Liang JH, Song YH, Xu Q, Chen XD, Mao LG, Li ZG (2017). The effects of second messenger cAMP and its relative components on the contraction of uterine smooth muscle of rat. Eur Rev Med Pharmacol Sci.

[CR62] Song H, Park J, Bui PTC, Choi K, Gye MC, Hong YC, Kim JH, Lee YJ (2017). Bisphenol A induces COX-2 through the mitogen-activated protein kinase pathway and is associated with levels of inflammation-related markers in elderly populations. Environ Res.

[CR63] Susiarjo M, Xin F, Bansal A, Stefaniak M, Li C, Simmons RA, Bartolomei MS (2015). Bisphenol a exposure disrupts metabolic health across multiple generations in the mouse. Endocrinology.

[CR64] Szymanska K, Gonkowski S (2018). Bisphenol A-induced changes in the enteric nervous system of the porcine duodenum. Neurotoxicology.

[CR65] Szymanska K, Makowska K, Gonkowski S. The influence of high and low doses of bisphenol A (BPA) on the enteric nervous system of the porcine ileum. Int J Mol Sci 2018, 19, pii: E917. doi: 10.3390/ijms19030917 10.3390/ijms19030917 PMC5877778 29558425

[CR66] Takahashi M, Komada M, Miyazawa K, Goto S, Ikeda Y (2018). Bisphenol A exposure induces increased microglia and microglial related factors in the murine embryonic dorsal telencephalon and hypothalamus. Toxicol Lett.

[CR67] Thoene M, Godlewski J, Rytel L, Dzika E, Bejer-Olenska E, Wojtkiewicz J. Alterations in porcine intrahepatic sympathetic nerves after bisphenol A administration. Folia Histochem Cytobiol 2018,a 1, 113–121. doi: 10.5603/FHC.a2018.0012, 56 10.5603/FHC.a2018.0012 29888781

[CR68] Viberg H, Lee I (2012). A single exposure to bisphenol A alters the levels of important neuroproteins in adult male and female mice. Neurotoxicology.

[CR69] Wang T, Xie C, Yu P, Fang F, Zhu J, Cheng J, Gu A, Wang J, Xiao H (2017). Involvement of insulin signaling disturbances in bisphenol A-induced Alzheimer’s disease-like neurotoxicity. Sci Rep.

[CR70] Xu X, Xie L, Hong X, Ruan Q, Lu H, Zhang Q, Zhang G, Liu X (2013). Perinatal exposure to bisphenol-A inhibits synaptogenesis and affects the synaptic morphological development in offspring male mice. Chemosphere.

[CR71] Yasuda K, Sumi G, Kanamori C, Nakajima T, Tsuzuki T, Cho H, Nishigaki A, Okada H, Kanzaki H (2014). Effects of ovarian hormone treatment on the gene expression of muscarinic acetylcholine receptors in the ovariectomized rat myometrium. J Steroid Biochem Mol Biol.

[CR72] Zhang L, Hancock JC, Hoover DB (2005). Tachykinin agonists modulate cholinergic neurotransmission at Guinea-pig intracardiac ganglia. J Pharmacol Sci.

[CR73] Zhang Q, Wu S, Liu L, Hou X, Jiang J, Wei X, Hao W (2018). Effects of bisphenol A on gap junctions in HaCaT cells as mediated by the estrogen receptor pathway. J Appl Toxicol.

